# Case Report: Probable hepatosplenic tuberculosis mimicking undifferentiated connective tissue disease and malignancy

**DOI:** 10.3389/fmed.2026.1959210

**Published:** 2026-10-28

**Authors:** Yongmei Han, Ning Shen, Qingqing Zhu, Mingli Ouyang, Ci Lu, Xian Wang

**Affiliations:** 1 Department of Rheumatology, Sir Run Run Shaw Hospital, Zhejiang University School of Medicine, Hangzhou, China; 2 Department of Radiology, Sir Run Run Shaw Hospital, Zhejiang University School of Medicine, Hangzhou, China; 3 Department of Medical Oncology, Sir Run Run Shaw Hospital, Zhejiang University School of Medicine, Hangzhou, China

**Keywords:** case report, diagnostic challenge, hepatosplenic tuberculosis, malignancy, undifferentiated connective tissue disease

## Abstract

**Background:**

Hepatosplenic tuberculosis is a rare form of extrapulmonary tuberculosis with nonspecific manifestations, often leading to misdiagnosis. Tuberculosis can mimic connective tissue disease by presenting with systemic symptoms and positive autoantibodies, posing a diagnostic challenge.

**Case presentation:**

We report a 69-year-old woman with polyarthralgia, oral ulcers, and positive autoantibodies including antinuclear antibody (ANA), anti-keratin antibody (AKA), and antiphospholipid antibodies, who was initially diagnosed with undifferentiated connective tissue disease (UCTD). Despite hydroxychloroquine treatment, she developed fever, weight loss, and neuropathy. Positron emission tomography-computed tomography (PET-CT) revealed multiple hypermetabolic hepatic and splenic lesions and abdominal lymphadenopathy, suspicious for malignancy. Liver biopsy demonstrated necrotizing epithelioid granulomas, which were highly suggestive of tuberculosis. In the context of a positive interferon-*γ* release assay, compatible imaging, consideration of relevant alternatives, and subsequent treatment response, probable hepatosplenic tuberculosis was considered the most likely diagnosis. Anti-tuberculosis therapy led to significant clinical and radiological improvement as assessed at six weeks.

**Conclusions:**

Our case highlights that probable hepatosplenic tuberculosis can mimic autoimmune diseases and malignancies. Biopsy is key to diagnosis, and treatment response may be supportive even when microbiology is negative.

## Introduction

Tuberculosis remains a major global health challenge and imposes a heavy disease burden. In a national survey in China, extrapulmonary tuberculosis (EPTB) without pulmonary involvement accounted for 21.3% of all tuberculosis cases (1). Hepatosplenic tuberculosis is relatively rare in EPTB and has nonspecific clinical manifestations, making it difficult to diagnose.

Patients with connective tissue diseases (CTD) are at increased risk of tuberculosis. Conversely, active tuberculosis can also be associated with the presence of autoantibodies (2, 3). When accompanied by positive autoantibodies and systemic symptoms, tuberculosis can closely mimic CTD, leading to misdiagnosis and unnecessary immunosuppressive therapy.

Herein, we report a patient with probable hepatosplenic tuberculosis supported by liver biopsy, who was initially misdiagnosed with undifferentiated connective tissue disease (UCTD) and later suspected of having malignancy. We present this case to highlight the diagnostic difficulty of probable hepatosplenic tuberculosis when transient autoantibody positivity and malignancy-like imaging obscure the underlying infectious process.

## Case presentation

A 69-year-old woman presented with a 1-year history of migratory joint swelling and pain that initially involved both knees with restricted movement and gradually extended to the right hip, both elbows, and the right wrist during the following months. The symptoms improved with symptomatic treatment but recurred repeatedly. She also reported multiple recurrent painful oral ulcers mainly on the tongue and palate, which resolved within days but recurred. She came to our department due to worsening symptoms. She had a 10-year history of hypertension well-controlled on amlodipine and a 10-year history of hyperthyroidism, for which antithyroid medication had been discontinued six months before her first visit, with subsequent normal thyroid function. She was a housewife with no history of tuberculosis exposure, no abnormal travel or residence history, no immunosuppressive state, no other medical history, and no smoking or alcohol use. She reported no history of fever, cough, rashes, recurrent miscarriages, thrombotic events or significant family history of autoimmune or thrombotic disorders. She was unsure whether she had received BCG vaccination in childhood, and no BCG scar was observed.

### Initial evaluation and diagnosis

Physical examination on admission showed swelling, tenderness, and restricted movement of both knees. In the other previously affected joints, there was mild tenderness with preserved movement. Vital signs including body temperature were normal, and body weight was 60 kg. No peripheral lymphadenopathy, hepatosplenomegaly, or skin rash was observed. Neurological examination was unremarkable. Laboratory tests revealed positive antiphospholipid antibodies including anticardiolipin antibody IgM (ACA-IgM) at 13.5 MPLU/mL, anti-β2-glycoprotein I antibody (anti-β2GP1) at 133.4 AU/mL, and lupus anticoagulant (LA) at 1.25. Other findings included positive antinuclear antibody (ANA) at 1:100 with a cytoplasmic granular pattern, positive anti-keratin antibody (AKA), mild pancytopenia, and significantly elevated inflammatory markers [erythrocyte sedimentation rate [ESR] at 89 mm/h and C-reactive protein [CRP] at 33.3 mg/L]. Further testing of the ANA profile, including anti-Ro/SSA, anti-U1 RNP, anti-dsDNA, and anti-Sm, were all negative. Anti-neutrophil cytoplasmic antibodies (ANCA) were also negative. Rheumatoid factor (RF), anti-cyclic citrullinated peptide antibody (anti-CCP), immunoglobulins, complement C3 and C4, liver and renal function, urinalysis, and tumor markers were within normal limits. Thyroid function tests were normal, and anti-thyroid peroxidase (anti-TPO), anti-thyroglobulin (anti-Tg), and TSH receptor antibody (TRAb) were all negative. QuantiFERON-TB Gold Plus (QB-SPOT) was positive. However, the patient denied having fever, night sweats, or weight loss at that time. Serological tests for hepatitis B, syphilis, and HIV were negative. Detailed laboratory data are summarized in Table 1 (M0). Ultrasound examination of the affected joints revealed no synovitis. Thyroid ultrasound showed a C-TIRADS 4A nodule, which was managed with follow-up. Ultrasound screening of the abdominal organs was normal. Additional imaging including ultrasound of the cervical, supraclavicular, and axillary lymph nodes, and non-contrast chest computed tomography (CT) was all unremarkable without active lesions.

**Table 1:** Laboratory results of the patient.

Months from 1st admission	M0	M10	M12	M13.5	Normal range
White blood cell, ×10^9^/L	**3**.**0**	**2.7**	**2.4**	**2.4**	3.5–9.5
Hemoglobin, g/L	**114**	**95**	124	130	115–150
Platelets, ×10^9^/L	**107**	**114**	**59**	**77**	125–350
Alanine transaminase, IU/L	15	9	12	10	7–40
Aspartate transaminase, IU/L	16	15	21	19	13–35
Creatinine, µmol/L	52	66	70	69	41–81
CRP, mg/L	**33**.**3**	**36.4**	2.4	2.0	0.0–6.0
ESR, mm/h	**89**	**114**	18	N/A	1–20
ANA	**1:100**	Negative	Negative	N/A	Negative
ANCA	Negative	N/A	N/A	N/A	Negative
ACA-IgG, GPLU/mL	7.4	2.5	3.8	N/A	a
ACA-IgA, APLU/mL	4.5	<2.5	2.5	N/A	a
ACA-IgM, MPLU/mL	**13**.**5**	6.3	7.6	N/A	a
Anti-*β*2GP1, AU/mL	**133**.**4**	**30.3**	**44**	N/A	b
Lupus anticoagulant	**1**.**25**	0.91	1.18	N/A	<1.2
AKA, RU/mL	**Positive**	**Positive**	**Positive**	N/A	Negative
RF, IU/mL	4.8	N/A	N/A	N/A	<14
Anti-CCP, U/mL	5	N/A	N/A	N/A	<5
IgA, g/L	3.83	4.37	3.47	N/A	0.82–4.53
IgG, g/L	14.9	14.5	14.1	N/A	7.51–15.6
IgM, g/L	1.18	1.13	1.05	N/A	0.46–3.04
Complement component 3, g/L	1.1	1.51	0.86	N/A	0.79–1.52
Complement component 4, g/L	0.28	0.27	0.19	N/A	0.16–0.38

a Reference range for ACA (all isotypes): <8 (negative); 8–12 (equivocal); >12 (positive).

b Reference range for anti-β2GP1: <16 (negative); 16–24 (equivocal); >24 (positive).

M0, M10, M12, M13.5: 0, 10, 12, 13.5 months from 1st admission.

Abnormal values are shown in bold.

CRP, C-reactive protein; ESR, erythrocyte sedimentation rate; ANA, antinuclear antibodies; ANCA, anti-neutrophil cytoplasmic antibodies; ACA, anticardiolipin antibodies; anti-β2GP1, anti-β2-glycoprotein I antibodies; AKA, anti-keratin antibodies; RF, rheumatoid factor; anti-CCP, anti-cyclic citrullinated peptide antibodies; Ig, immunoglobulin; N/A, not available.

The patient was provisionally diagnosed with UCTD as a working diagnosis, based on recurrent oral ulcers, polyarthralgia, mild pancytopenia, positive autoantibodies (ANA, antiphospholipid antibodies, and AKA), and elevated inflammatory markers. As the Mosca preliminary classification criteria for UCTD require a disease duration of at least three years (4), the patient's diagnosis was therefore considered provisional. Given the positive QB-SPOT result, active tuberculosis was also considered at this stage, but the absence of constitutional symptoms and the unremarkable chest CT made it less likely. Hydroxychloroquine (HCQ) was initiated as initial therapy at a dose of 200 mg twice daily.

### Progression and re-evaluation

The patient was followed up at the outpatient department. One month after initiating HCQ, her joint symptoms improved with partial recovery of blood counts. ESR decreased to 39 mm/h and CRP to 16.8 mg/L. Although she remained asymptomatic with normal blood counts over the following six months, her ESR and CRP gradually increased (Figure 1A). She was readmitted ten months later due to relapsing polyarthralgia, progressive numbness in both feet, and patient-reported intermittent chills and fever with a maximum documented temperature of 37.6 ℃. Additionally, she reported a weight loss of 7 kg in the past year. On readmission, her body weight was 53 kg. Neurological examination showed decreased vibration and pinprick sensation over both feet, with preserved muscle strength and reflexes. Electromyography showed peripheral neuropathy with predominant sensory nerve involvement. Laboratory tests revealed elevated ESR at 114 mm/h and CRP at 38.8 mg/L, along with mild leukopenia, anemia, and thrombocytopenia (Figures 1A,B). Her previously positive autoantibodies, including ACA-IgM, LA, and ANA had all turned negative, with the exception of a low-titer anti-β2GP1 antibody at 30.3 AU/mL and AKA positivity (Table 1, M10).

**Figure 1:**
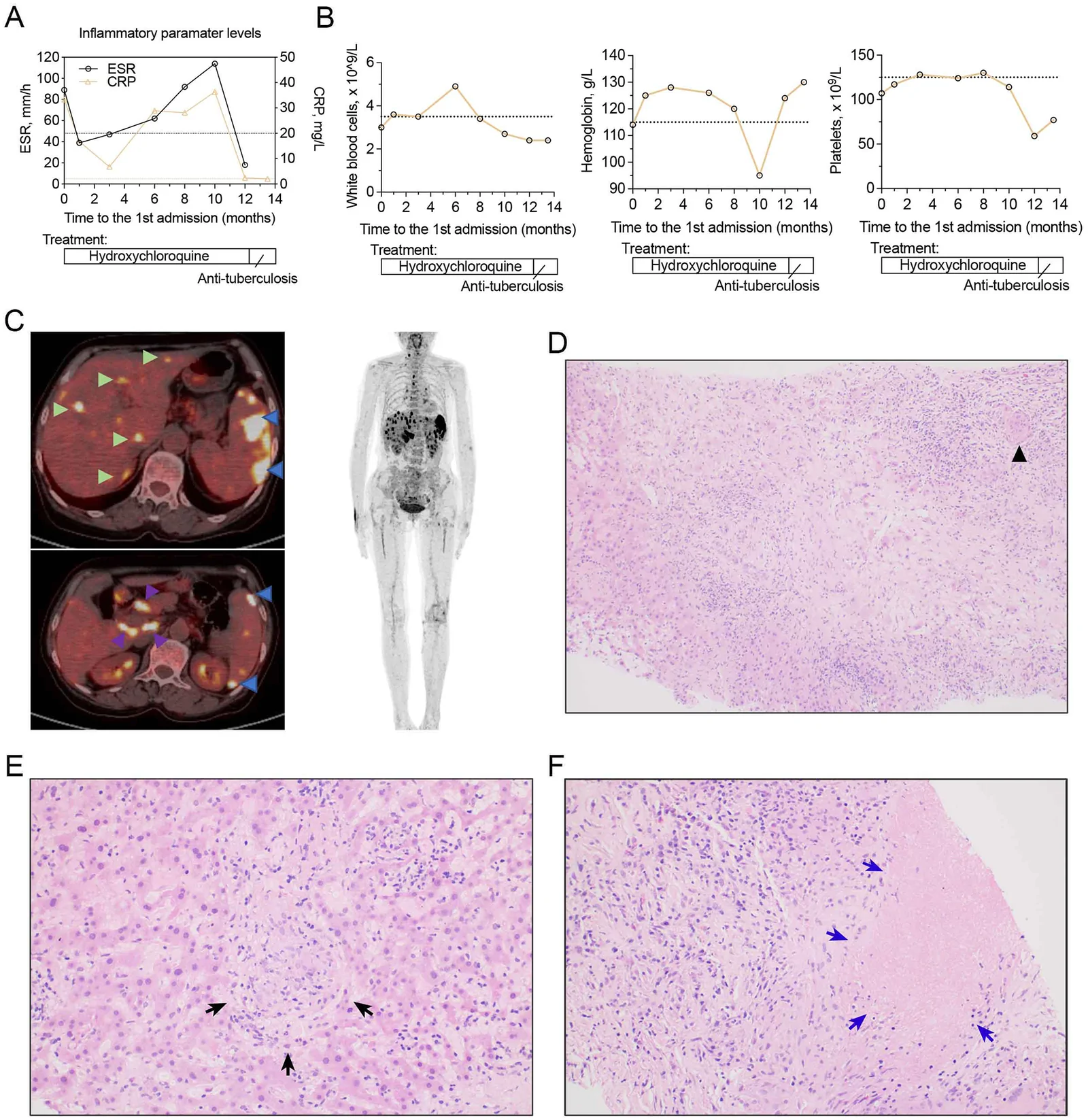
Clinical, imaging, and histopathological findings of the patient. **(A)** Trends of ESR and CRP during follow-up. **(B)** Trends of white blood cell, hemoglobin, and platelet counts during follow-up. **(C)** PET-CT images showing multiple hypermetabolic lesions in the liver (green triangles) and spleen (blue triangles), and multiple enlarged abdominal lymph nodes with increased FDG uptake (purple triangles). **(D)** Liver biopsy showing destroyed hepatic architecture with fibrous tissue proliferation, a small granuloma (black triangle) in the upper right corner, and dilated sinusoids in the residual liver tissue on the left. **(E)** Epithelioid granuloma (black arrows) in a hepatic lobule. **(F)** Epithelioid granuloma with central coagulative necrosis (blue arrows). **(D–F)** Hematoxylin and eosin (HE) staining. ESR, erythrocyte sedimentation rate; CRP, C-reactive protein; PET-CT, positron emission tomography-computed tomography; FDG, fluorodeoxyglucose.

On readmission, the patient presented with relapsing polyarthralgia, fever, peripheral neuropathy, significant weight loss, and markedly elevated inflammatory markers, raising concern for an occult malignancy or systemic vasculitis. Therefore, an F-18 fluorodeoxyglucose (FDG) positron emission tomography-computed tomography (PET-CT) was performed as an early investigation to identify hypermetabolic lesions and a suitable biopsy site. Her PET-CT revealed multiple hypermetabolic lesions in the liver and spleen, along with multiple enlarged lymph nodes in the hepatogastric space, hepatic hilum, portocaval space, and retroperitoneum (Figure 1C). The maximum standardized uptake values (SUVmax) were 11 for the hepatic lesions, 24 for the splenic lesions, and 18 for the involved lymph nodes. Contrast-enhanced upper-abdominal magnetic resonance imaging (MRI) further confirmed these findings (Figures 2A–C). Based on these imaging findings, malignancy or lymphoma was highly suspected and a liver biopsy was performed immediately. Unexpectedly, no malignant cells were identified on histopathological examination. Instead, several epithelioid granulomas were observed, one of which contained necrosis (Figures 1D–F). Although acid-fast (AF) staining, periodic acid-Schiff (PAS) staining, and tuberculosis-DNA (TB-DNA) testing were all negative, tuberculosis remained the primary consideration based on the histopathological findings, the patient's symptoms of fever, weight loss, and neuropathy, and the positive QB-SPOT result. In this patient, other potential infectious and non-infectious causes of necrotizing granulomas were also considered, including non-tuberculous mycobacteria (NTM), fungal infections, rheumatoid nodules, and granulomatosis with polyangiitis (GPA). However, rheumatoid nodules and GPA were less likely based on the clinical picture and the absence of features of systemic arthritis or vasculitis. To obtain microbiological confirmation and to evaluate for NTM and fungal infections, we recommended a repeat liver biopsy for culture, Xpert MTB/RIF, and next-generation sequencing (NGS), but the patient declined further invasive procedures. Consequently, a triple anti-tuberculosis regimen comprising rifampicin 450 mg once daily, isoniazid 300 mg once daily, and levofloxacin 500 mg once daily, all administered orally, was initiated as a diagnostic and therapeutic trial under the guidance of an infectious disease specialist. A timeline of the patient's clinical course is presented in Figure 3.

**Figure 2:**
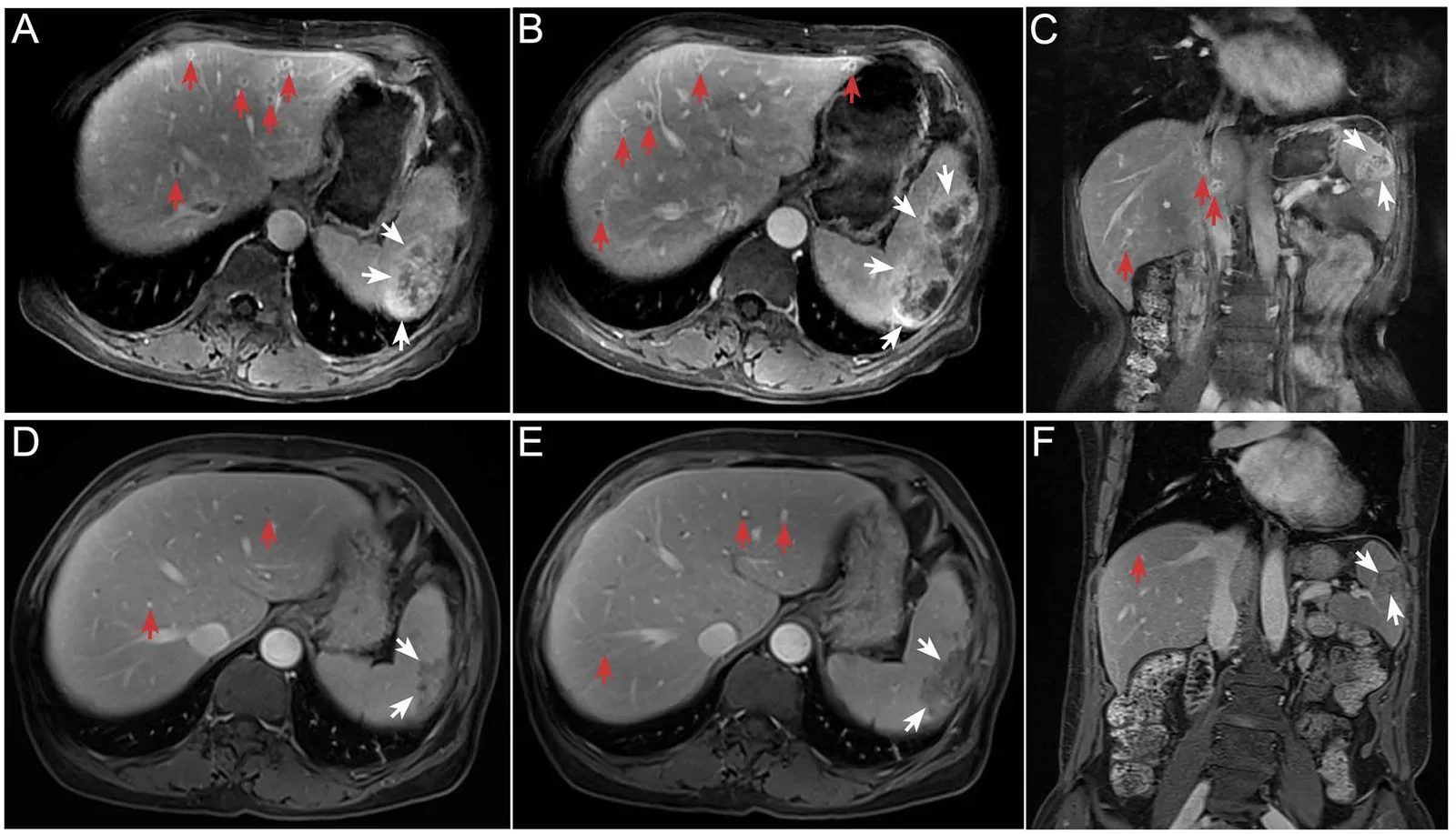
Abdominal contrast-enhanced MRI findings before and after anti-tuberculosis therapy. **(A–C)** Pre-treatment images showing multiple nodular and mass-like lesions in the liver (red arrows) and spleen (white arrows) with heterogeneous sizes, characterized by low T1 and high T2 signal intensity, with rim enhancement after contrast administration. **(D–F)** Follow-up images after six weeks of anti-tuberculosis therapy demonstrating partial resolution or decrease in size of the hepatic (red arrows) and splenic (white arrows) lesions. MRI, magnetic resonance imaging; T1, T1-weighted; T2, T2-weighted.

**Figure 3:**
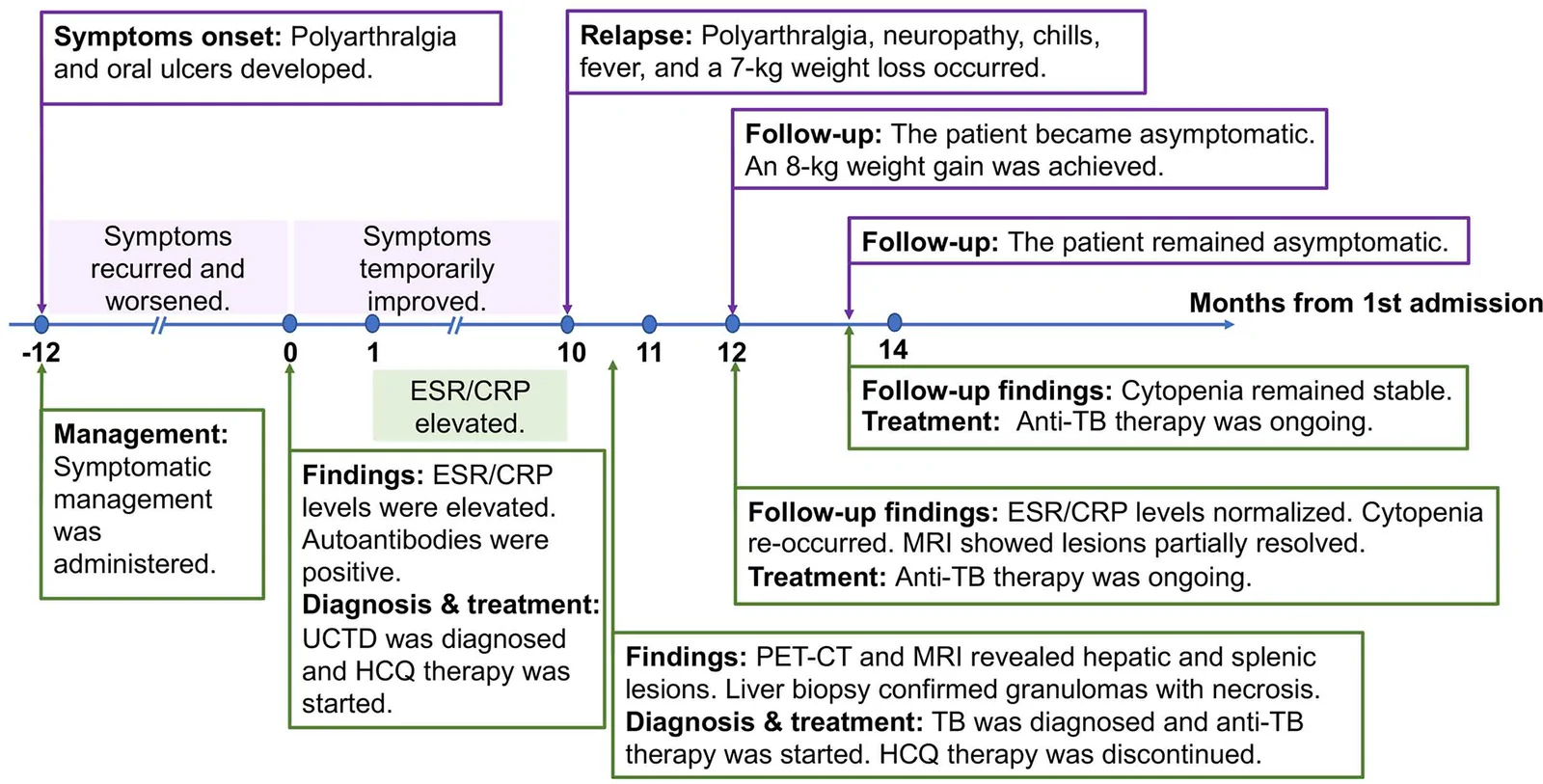
Timeline of the patient's clinical course, diagnostic evolution, and therapeutic response. UCTD, undifferentiated connective tissue disease; HCQ, hydroxychloroquine; ESR, erythrocyte sedimentation rate; CRP, C-reactive protein; TB, tuberculosis; PET-CT, positron emission tomography-computed tomography; MRI, magnetic resonance imaging.

### Treatment and follow-up

After six weeks of anti-tuberculosis therapy, the patient returned for a follow-up evaluation. She was asymptomatic, reporting no joint symptoms, no fever, no numbness, and had gained 8 kg in body weight. The clinical and radiological improvement observed after anti-tuberculosis therapy further supported the diagnosis of probable hepatosplenic tuberculosis. Laboratory tests showed normalization of ESR and CRP (Figure 1A). Anemia had resolved, while worsening leukopenia (2.4 × 10^9^/L) and thrombocytopenia (59 × 10^9^/L) had developed (Figure 1B), which were considered possibly treatment-associated based on their temporal relationship to treatment initiation. Follow-up contrast-enhanced MRI demonstrated that the abnormal hepatic and splenic lesions had partially resolved or decreased in size (Figures 2D–F). Of note, HCQ had been discontinued upon initiation of anti-tuberculosis treatment, and her autoantibodies including ANA, ACA-IgM, and LA remained negative. Anti-*β*2GP1 antibody was still positive with a titer of 44 AU/mL and AKA remained positive (Table 1, M12). Given the favorable clinical and radiological response, the regimen was continued with close monitoring. After a further six weeks, the leukocyte count was 2.4 × 10^9^/L, hemoglobin 130 g/L, and platelet count 77 × 10^9^/L, with a CRP of 2.0 mg/L. The patient remained asymptomatic, and treatment was continued without dose adjustment or additional intervention. Follow-up imaging and autoantibody testing were not repeated at this visit (Table 1, M13.5).

## Discussion

In this report, we describe a patient who initially presented with polyarthralgia, oral ulcers, positive autoantibodies, and elevated inflammatory markers, leading to a provisional diagnosis of UCTD. Despite HCQ treatment, she developed progressive symptoms, and imaging revealed multiple lesions in the liver and spleen suspicious for malignancy. Liver biopsy unexpectedly revealed necrotizing epithelioid granulomas which were highly suggestive of tuberculosis despite the absence of microbiological confirmation. In addition, the patient's favorable response to anti-tuberculosis treatment further supported the diagnosis of probable hepatosplenic tuberculosis.

In this case, a major turning point during follow-up came when the patient developed fever and significant weight loss, and the subsequent PET-CT revealed multiple hypermetabolic lesions in the liver and spleen, along with extensive hypermetabolic lymphadenopathy in the abdominal cavity. Initially, given her autoimmune disease background and the imaging features, we preferentially suspected malignancy. First, the risk of malignancy is known to be increased in patients with autoimmune diseases (5, 6). Second, isolated hepatic and splenic tuberculosis is rare, making it easily overlooked. Third, the patient's symptoms of fever and weight loss were also highly suggestive of malignancy. However, subsequent liver biopsy revealed necrotizing epithelioid granulomas. Despite the negative AF staining and TB-DNA testing, tuberculosis was considered based on the combination of histopathological findings, clinical symptoms, and the positive QB-SPOT result. Extrapulmonary tuberculosis is often paucibacillary, so negative staining or nucleic-acid testing does not exclude the diagnosis. The low microbiological yield of liver biopsy in hepatic tuberculosis has been documented. Reported sensitivities range from 25% for AF staining in a systemic review of 618 patients to 60% in another review, while histology and PCR have higher sensitivities (7, 8). Empiric treatment should therefore integrate tissue findings, imaging, epidemiological risk, exclusion of competing diagnoses, and objective follow-up (9). In addition, the differential diagnosis of infectious and non-infectious causes, such as non-tuberculous mycobacteria, fungal infections, rheumatoid nodules, and GPA should be considered (10). Although the patient had polyarthralgia and positive AKA, rheumatoid arthritis and rheumatoid nodules were unlikely because her RF and anti-CCP were negative and no synovitis was observed on ultrasound of her joints. GPA was considered less likely because of the absence of systemic vasculitic symptoms and negative ANCA.

Several similar cases have been reported in which hepatosplenic tuberculosis presented as multiple micronodular and macronodular lesions on CT or MRI (11, 12), as well as hypermetabolic lesions on PET-CT (13, 14), which are consistent with our findings. In addition, in rare cases, hepatosplenic tuberculosis may present as diffuse FDG uptake in the liver and spleen on PET-CT (15, 16). However, many etiologies including hepatosplenic metastasis, lymphoma, sarcoidosis, and candidiasis can share similar PET-CT findings (17–19). The intense FDG uptake in tuberculous granulomas is attributed to the metabolic activity of activated macrophages and lymphocytes, which also express high levels of GLUT-1 glucose transporters through a mechanism similar to that in malignant cells (20). This explains why hepatosplenic tuberculosis can closely mimic lymphoma or metastatic disease on PET-CT. Therefore, tissue biopsy plays a critical role in supporting the diagnosis of hepatosplenic tuberculosis when imaging findings are ambiguous or misleading.

The anti-tuberculosis regimen was chosen as an individualized deviation from the standard regimen. It was determined by an infectious diseases specialist after weighing the risk of drug toxicity against the need for an adequate multidrug regimen. As the diagnosis was not microbiologically confirmed, the regimen was intended as a diagnostic and therapeutic trial, and safety was prioritized to ensure tolerability. Levofloxacin was preferred as a substitute based on its anti-tuberculous activity. Ethambutol was avoided because of the patient's advanced age and history of hypertension, both of which are established risk factors for ethambutol-induced optic neuropathy (21, 22), although these are not absolute contraindications. Pyrazinamide was also avoided because of the potential risk of hepatotoxicity in the setting of hepatic involvement (23), although liver enzymes were normal and hepatic involvement alone does not establish a contraindication. Although a standard 6-month treatment is generally considered sufficient for most forms of extrapulmonary tuberculosis (14, 24), prolonged therapy of 12 months or even 24 months has also been reported in some cases of splenic tuberculosis (25, 26). In our patient, drug resistance could not be formally assessed because of the absence of culture and susceptibility testing, and the regimen represented a nonstandard, individualized deviation from standard care. Therefore, the planned duration was extended to 12 months, with adjustment based on clinical and radiological response.

A notable feature of this case is the evolution of autoantibody profiles. At initial presentation, the diagnosis of UCTD appeared reasonable. Several features supported this diagnosis. First, the patient presented with polyarthralgia, oral ulcers, and pancytopenia, which were commonly seen in UCTD patients at the time of diagnosis (27). Arthralgia was one of the most frequent symptoms occurring in more than 80% of patients (27). Second, there was serological evidence of autoimmunity including positive ANA, antiphospholipid antibodies, and AKA, along with significantly increased ESR and CRP levels. Other conditions were nevertheless considered. Systemic lupus erythematosus (SLE) was considered unlikely because anti-dsDNA and anti-Sm were negative and complement levels were normal. Rheumatoid arthritis (RA) was considered unlikely because RF and anti-CCP were negative and joint ultrasound showed no synovitis. Antiphospholipid syndrome (APS) was considered less likely because there was no history of thrombosis or miscarriage. Sjögren's syndrome (SS) and mixed connective tissue disease (MCTD) were considered unlikely because anti-Ro/SSA and anti-U1 RNP were negative. Although the patient had a history of hyperthyroidism, autoimmune thyroid disease was also considered less likely given the normal thyroid function, and the negative anti-TPO, anti-Tg, and TRAb. At the initial presentation, the infectious workup was limited because the patient had no fever or localized infectious symptoms. She presented primarily with polyarthralgia and oral ulcers, which are more typical of connective tissue disease. Additionally, despite the positivity of QB-SPOT, active tuberculosis was considered less likely at that time as the patient did not report any fever, night sweats, or weight loss at that time, and we found no further clues of active tuberculosis after screening of the lung, abdominal organs, and superficial lymph nodes. Although, the diagnosis of UCTD requires a disease duration of at least 3 years (4, 28), it was considered a provisional working diagnosis, and we initiated HCQ given her prominent clinical symptoms and elevated inflammatory markers. However, several features observed during follow-up, including the lack of sustained response to hydroxychloroquine, the gradual rise in inflammatory markers, and the eventual emergence of fever, peripheral neuropathy, and significant weight loss, prompted us to reconsider the initial diagnosis.

By the time of the patient's re-admission (M10), ANA, ACA-IgM, and LA had already turned negative, before anti-tuberculosis treatment was initiated, while AKA remained positive and anti-*β*2GP1 remained positive at a reduced level. Active tuberculosis can also be associated with the production of a wide range of autoantibodies, including ANA and antiphospholipid antibodies (2, 3). Elkayam et al. reported that 30% of active tuberculosis patients had elevated anti-β2GP1 antibodies, and both anti-β2GP1 and anticardiolipin antibodies normalized after treatment (29). Similarly, a Brazilian study demonstrated that antiphospholipid antibodies, including ACA-IgM, were significantly elevated in active pulmonary tuberculosis patients and decreased after treatment (30). However, the change in autoantibodies in our patient cannot be attributed to anti-tuberculosis treatment, because her ANA, ACA-IgM, and LA had already turned negative before treatment was initiated. This case therefore illustrates that the autoantibody profile may be infection-associated, but it does not demonstrate anti-tuberculosis treatment-related normalization.

Beyond autoantibody production, the patient's polyarthralgia may also be explained by tuberculosis-related immune dysregulation. In particular, tuberculosis can induce a reactive arthritis known as Poncet's disease, which is characterized by polyarthritis or polyarthralgia without direct mycobacterial invasion of the joint. Its pathogenesis is considered immune-mediated. Proposed mechanisms include molecular mimicry between mycobacterial heat-shock proteins and human cartilage proteoglycans, deposition of circulating immune complexes in the synovium, and T-cell-mediated cross-reactivity (31, 32). A systematic review of 198 cases found that the most commonly affected joints were the ankles, knees, wrists, and elbows, with no axial involvement and joint symptoms typically resolve after anti-tuberculous therapy without chronicity (31). A site of tuberculosis infection was specified in 96.5% of cases, of which 56.8% were extrapulmonary (31). In our patient, the mainly involved joints included the knees, elbows, and right wrist, all of which are among the most commonly affected joints in Poncet's disease. Furthermore, the joint symptoms resolved after anti-tuberculosis therapy without any anti-rheumatic treatment, further supporting that the polyarticular symptoms were related to tuberculosis infection.

Several limitations of this case should be acknowledged. First, when the patient was found to have a positive QB-SPOT at initial evaluation, a tuberculin skin test (PPD) was not performed despite negative screening imaging. This may have represented a missed opportunity to further evaluate the possibility of latent or active tuberculosis at an earlier stage. Second, the liver biopsy revealed necrotizing epithelioid granulomas, which were nonspecific, and alternative causes could not be completely excluded. Furthermore, a broader infectious workup, including blood culture and serologies for other pathogens such as fungal and Brucella was not performed because the patient had no high fever or localized infectious symptoms. These unperformed investigations may have limited the exclusion of alternative etiologies. Third, microbiological confirmation and drug susceptibility data were unavailable, and the anti-tuberculosis therapy was nonstandard and incomplete with a follow-up period of only 12 weeks. Both treatment efficacy and safety remain to be confirmed by further follow-up. Nevertheless, the patient's favorable clinical and radiological response to anti-tuberculosis therapy strongly supports the diagnosis of probable hepatosplenic tuberculosis, and a therapeutic trial is often considered acceptable in culture-negative cases with compatible histopathological findings. Despite these limitations, this case provides longitudinal data on the evolution of autoantibody profiles and imaging findings, which may help clinicians recognize probable hepatosplenic tuberculosis when it mimics autoimmune disease.

In conclusion, probable hepatosplenic tuberculosis can mimic autoimmune diseases and malignancies. Clinicians should suspect it in patients with unexplained systemic inflammation and hepatic lesions, particularly when immunosuppressive therapy is ineffective. Biopsy is important, but when microbiological confirmation is unavailable, histopathology and treatment response support rather than confirm the diagnosis after excluding alternatives.

## Patient perspective

The patient reported that she experienced significant frustration during the diagnostic process. She initially believed her joint pain and oral ulcers were related to a rheumatic condition and felt confused when the diagnosis later changed to tuberculosis. She was concerned when malignancy was suspected based on the PET-CT findings, and felt relieved when the biopsy suggested probable hepatosplenic tuberculosis rather than cancer. She noted that her symptoms improved substantially after starting anti-tuberculosis treatment, and she was able to regain weight and resume normal daily activities. She expressed appreciation for the medical team's efforts to establish a definitive diagnosis.

## Data Availability

The raw data supporting the conclusions of this article will be made available by the authors, without undue reservation.
